# Comparative Leaf Proteome Analysis of Maize (*Zea mays* L.) Exposed to Combined Drought and Heat Stress

**DOI:** 10.3390/plants14223419

**Published:** 2025-11-08

**Authors:** Cleopatra Pfunde, Charles Shelton Mutengwa, Graeme Bradley, Nyasha Esnath Chiuta

**Affiliations:** 1 Department of Agronomy, Faculty of Science and Agriculture, University of Fort Hare, Alice 5700, South Africacmutengwa@ufh.ac.za (C.S.M.); 2 Department of Biochemistry and Microbiology, University of Fort Hare, Alice 5700, South Africa

**Keywords:** combined stress, climate-resilient, physiological traits, proteomics

## Abstract

This study sought to screen 45 maize (*Zea mays* L.) inbred lines for tolerance to combined drought and heat stress (CDHS) and identify the leaf proteome patterns of two inbred lines with contrasting stress response at early vegetative stage. Biomass accumulation was significantly reduced under CDHS compared to optimum conditions. Furthermore, CDHS-tolerant inbred lines exhibited significantly lower (*p* < 0.05) leaf temperatures (28.6 °C) and higher sub-stomatal CO_2_ concentration (9012 mol mol^−1^) and photosynthetic yield (0.69) under stress. The tolerant (CIM18) and susceptible (QS21) inbred lines were exposed to stress by maintaining a field capacity of 25% for 7 days and increasing the daily ambient temperature by 5 °C from 25 °C to 40 °C. Conventional two-dimensional electrophoresis analysis was used to compare leaf protein expression profiles, and significant differences (*p* < 0.05) were observed. Out of a total of 505 proteins, 114 showed significant quantitative variation. Of these, 62 proteins had a twofold upregulation in CIM18, while 52 were downregulated. Twenty upregulated proteins were selected for amino acid micro-sequencing, and 11 proteins were uniquely expressed in CIM18. The other nine proteins had ≥ twofold upregulation in CIM18 compared to QS21. The functions of the identified proteins included defence, metabolism, photosynthesis and structure.

## 1. Introduction

Maize (*Zea mays*) is the third most important cereal crop and plays a significant role in economic development and global food security [1]. However, its production encounters a wide array of abiotic stressors, such as combined drought and heat stress (CDHS). Maize is highly sensitive to severe CDHS at early vegetative stages, which can greatly reduce total crop stand and increase its vulnerability to pests and diseases [2]. Grzesiak et al. [3] reported a close correlation between maize seedling growth and grain yield under drought stress. Combined drought and heat stress presents a unique set of challenges, as the simultaneous occurrence of these conditions can exacerbate the negative effects of each stress on maize physiology. These stressors can cause protein dysfunction, which severely impacts maize growth and development, subsequently reducing grain yield [4]. This necessitates the development of tolerant varieties, especially, in the Sub-Saharan region, where both stressors are quite prominent early in the season.

Adaptability to drought or heat stress in maize has been attributed to various physiological traits such as photosynthetic efficiency, stomatal conductance, leaf water potential and antioxidant enzyme activity [4]. By investigating these physiological variables, researchers can gain insights into the mechanisms underlying maize’s resilience to CDHS. This knowledge is essential for developing strategies to improve maize tolerance and ensure sustainable agricultural productivity in the face of climate change.

Generally, plants respond uniquely to the occurrence and severity of abiotic stressors at different stages of crop growth, thereby increasing the complexities of breeding for climate-resilient varieties. However, different omics approaches, such as transcriptomics, metabolomics, phenomics, proteomics, etc., have significantly advanced smart breeding programs [5]. The proteomic approach is an important technique which provides great perceptions and understanding of plant responses to abiotic stressors at the protein level [6], capturing cell activity at a given time [5]. This method focuses on the actively translated portion of the genome, thus providing information missing in deoxyribonucleic acid (DNA) or messenger RNA analysis methods [7].

Proteomic analysis has been used to screen genotypes for drought and/or heat stress tolerance [6]. Comparative protein analysis has been conducted in maize [8], wheat [9], soybean [10] and several field crops exposed to drought stress [11,12]. Limited water stress can result in modification of several proteins such as aquaporins, dehydrins, heat shock proteins and late embryogenesis abundant (LEA) proteins [6]. Dehydrins are known to accumulate during late embryogenesis and on vegetative tissues of stressed (i.e., via drought, heat, salinity and cold) plants. They are therefore a group of LEA proteins that are synthesized more abundantly under stress and induce accumulation of abscisic acid (ABA). There is substantial evidence in the literature that shows how the accumulation of dehydrins confers tolerance to several abiotic stressors, including drought [13].

Heat stress influences the structure of cell membranes and physiological processes such as respiration and photosynthesis [14]. When temperatures increase, normal protein synthesis declines and stress-related proteins such as heat shock proteins (HSPs) accumulate, allowing the plant to adjust, which is necessary for conferring tolerance [15]. These proteins can be classified into five families based on their molecular masses: HSP100, HSP90, HSP70, HSP60 and small HSPs. The more abundant and diverse the HSPs, the more adapted a plant is to heat stress [16].

Proteomic analysis is increasingly becoming a biomarker of choice in marker-assisted selection (MAS) studies, where the unique protein associated with a trait is used to identify tolerant genotypes [17]. Proteomic research into maize plants subjected to individual abiotic stressors is well documented, but far fewer investigations have been conducted under combined stresses [18]. Previous proteomic studies have indicated that gene expression maybe altered, resulting in protein expression changes in plants subjected to heat and drought stress.

Over the years, a lot of technological advancement with regard to proteomic analysis, from conventional gel electrophoresis to label-free protein identification, has been observed [19]. Several proteomic technologies, such as mass spectrometry, protein arrays, affinity proteomics, ELISA and Western blot, have been developed [20]. However, the separation of expressed proteins using the convectional two-dimensional electrophoresis method is still useful. Proteomics relies on readily available genome sequence data to identify proteins of interest. Where sequences are not available, the proteins can be identified through similarity searches using homologous proteins from closely related species. Therefore, the objective of this study was to screen maize inbred lines for tolerance to CDHS and to identify proteins associated with maize tolerance at the seedling stage.

## 2. Results

### 2.1. Effect of Combined Drought and Heat Stress on Physiological Traits of Maize and Biomass

#### 2.1.1. Analysis of Variance

Sub-stomatal CO_2_ (C_i_), leaf temperature (T_leaf_), photosynthetic yield (PSII) and shoot and root dry weight were highly significantly different (*p* < 0.001) among inbred lines exposed to CDHS (Table A1).

#### 2.1.2. Sub-Stomatal CO_2_

Combined drought and heat stress exhibited a significant (*p* < 0.001) increase in CO_2_ uptake compared to optimum conditions. Inbred line CIM 20 recorded the highest sub-stomatal CO_2_ of 9012 mol mol^−1^ under CDHS (Table 1). However, QS21 recorded significantly lower sub-stomatal CO_2_ (531 mol mol^−1^) and was part of the bottom five lines that showed great sensitivity to combined stress (Table 1). On the other hand, CIM 18 showed moderate tolerance by recording a sub-stomatal CO_2_ of 3403 mol mol^−1^.

#### 2.1.3. Leaf Temperature

Leaf temperature was significantly (*p* ≤ 0.05) elevated by 3–8 °C in plants exposed to CDHS compared to the control. Stressed plants continued to show higher leaf temperatures throughout the experiment. Inbred lines CIM12 and CIM18 had the lowest leaf temperatures under stresses of 28.3 °C and 28.6 °C, respectively (Table 2). Contrariwise, sensitive lines recorded leaf temperatures above 35 °C.

#### 2.1.4. Photosynthetic Yield (PSII)

Efficiency of photosynthetic yield was significantly (*p* < 0.001) higher in control plants compared to those under stressed conditions throughout the experiment. Photosynthetic yield efficiency was 17–29% less under CDHS than in control plants. Inbred lines QS17 and QS1 had the highest photosynthetic yield during stress, closely followed by QS30, QS16 and CIM 18 (Table 2). After the recovery period, differences in PSII efficiency were non-significant (*p* > 0.05).

#### 2.1.5. Shoot and Root Dry Weight

Shoot and root dry weight were significantly reduced by CDHS. Inbred lines CIM 12, QS22 and CIM 18 were the top three best performers for shoot and root dry weight under CDHS. Inbred line QS 21 recoded the lowest dry shoot weight (0.07 g) and moderated dry root weight (0.05 g) under stress. Generally, shoots were more severely affected by stress compared to roots.

Although the primary focus was on stress-induced changes, the inclusion of a 3-day recovery period allowed for assessment of resilience. Notably, PSII efficiency differences between CIM18 and QS21 were no longer significant post-recovery, indicating partial restoration of photosynthetic function. The higher final biomass and upregulation of stress-responsive proteins in CIM18 suggest a more robust recovery mechanism compared to QS21. These findings highlight the importance of recovery capacity as a component of stress tolerance.

#### 2.1.6. Stress Tolerance Indices

Shoot dry weight was used to calculate different indices, namely stress tolerance index (STI), drought resistance index (DRI) and modified stress tolerance index (K_2_STI). Based on the results, QS22, QS6 and CIM18 exhibited CDHS tolerance, as indicated by the higher index values recorded (Table 2).

#### 2.1.7. Quantitative Comparative Analysis of Protein Responses

**Figure 1:**
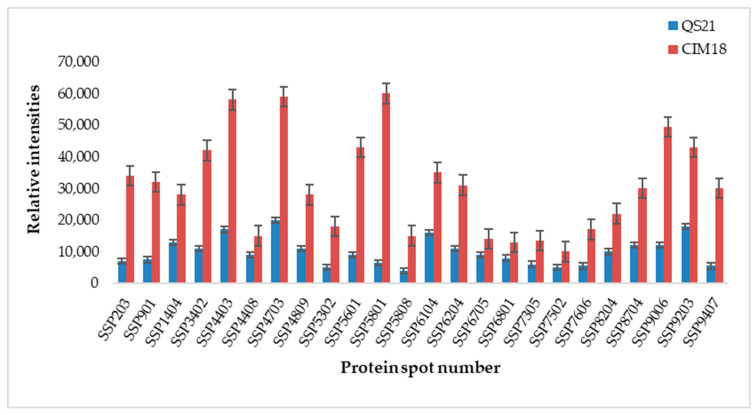
Twenty-four protein spots which significantly increased twofold or more in the tolerant (CIM18) compared to susceptible (QS21) inbred line under combined drought and heat stress treatment. Data shown are means ± SD of three biological replicates.

Comparative proteomic analysis was used to investigate the change in protein profiles in leaves of tolerant (CIM18) and susceptible (QS21) maize inbred lines after exposure to CDHS. Thus, this study focused on comparative assessment of proteins differentially expressed by these two inbred lines under CDHS. Approximately 505 spots were reproducibly detected in each Sypro Ruby-stained gel. The partial least squares analysis revealed that 185 protein spots were significantly different (*p* < 0.05) between CIM18 and QS21. Of these, 114 proteins exhibited a twofold differential expression (increase/decrease) between the tolerant and susceptible inbred lines. A total of 62 of these were either upregulated or newly induced in response to CDHS, while 52 were downregulated in CIM18. Of the 114 proteins, QS21 had 52 proteins upregulated and 62 downregulated. Twenty-four protein spots that showed a twofold increase or more in CIM18 and exhibited a coefficient of variation between 0% and 2% are shown in Figure 1.

**Figure 2:**
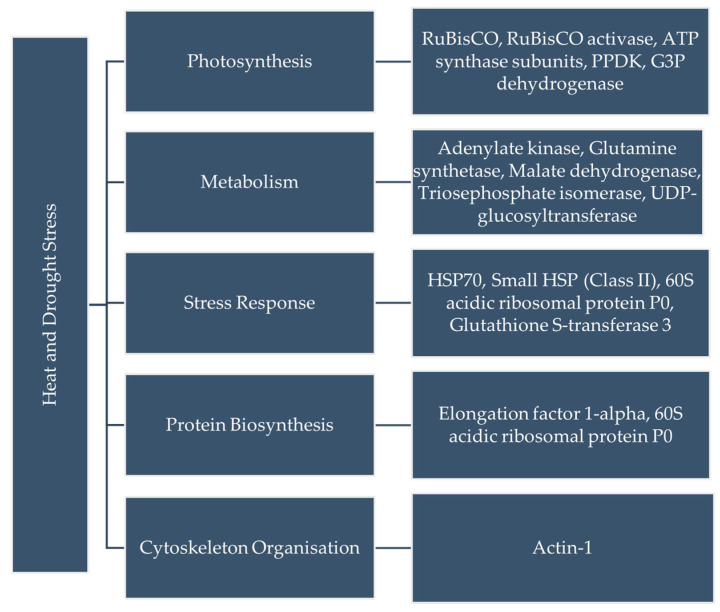
Functional categories of proteins uniquely upregulated in the combined drought and heat stress-tolerant inbred line, CIM18.

**Figure A1:**
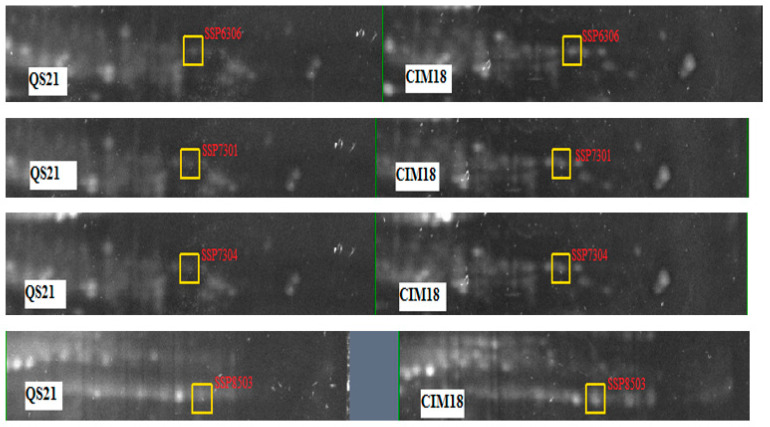
Gel images of protein spots occurring in maize leaves of both inbred lines, QS21 (susceptible) and CIM18 (tolerant), under combined drought and heat stress. Prominent spots indicate upregulation, and vice versa.

Examples of gel pictures showing protein upregulation are shown in Figure A1, whereas the functional categories of proteins uniquely upregulated in the CDHS-tolerant inbred line CIM18 are shown in Figure 2.

**Figure 3:**
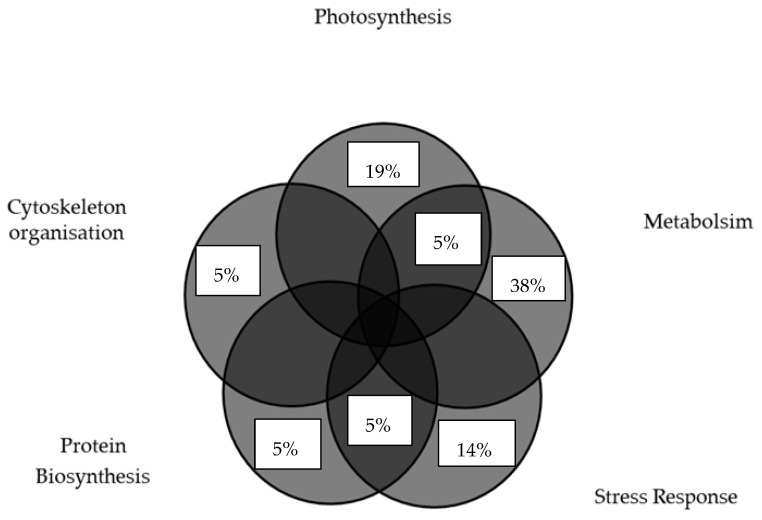
Percentage of proteins uniquely upregulated in the combined drought and heat stress-tolerant inbred line, CIM18, according to functional category.

**Figure A2:**
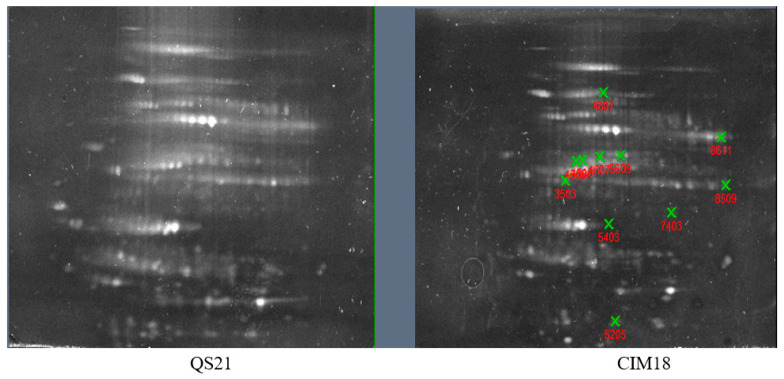
Representative 2-DE gel of susceptible QS21 and tolerant CIM18 lines under combined drought and heat stress. The green crosses show differentially expressed spots occurring in CIM18 but not in QS21.

The flow chart provides a clear overview of how the proteins were grouped into broader categories: photosynthesis, metabolism, stress response, protein biosynthesis and cytoskeleton organization. Eleven protein spots were unique only to the tolerant inbred line (CIM18), as shown in Figure A2. The percentage of proteins uniquely upregulated in the tolerant CIM18 inbred line is shown in Figure 3.

The identified spots were classified according to their putative functions. The differentially expressed proteins and their individual functions are detailed in Table 3.

## 3. Discussion

Plants exhibit stress tolerance or avoidance through acclimation and adaptation mechanisms. This study identified QPM inbred lines that exhibited tolerance to CDHS using physiological traits such as gaseous exchange, photosynthetic yield and leaf temperature. A wide genetic variation existed among the evaluated inbred lines. Similar screening and characterization studies are useful in preliminary elimination of susceptible lines in breeding programs involving several genotypes.

Generally, drought reduces internal CO_2_ due to stomata closure, whereas heat stress increases sub-stomatal CO_2_ concentration due to reduced efficacy of ribulose bisphosphate carboxylase/oxygenase (RUBISCO) activase caused by elevated leaf temperatures [21]. As such, CDHS can cause complexities in overall sub-stomatal CO_2_ concentration [22]. In this study, CIM 18 exhibited higher sub-stomatal CO_2_ concentration relative to QS21. Additionally, CIM 18 recorded lower leaf temperature and higher photosynthetic yield. Similarly, the ability to balance between conserving water by closing the stomata or opening the stomata to allow transpirational cooling and CO_2_ influx was observed on CDHS-tolerant broadleaf evergreen shrubs [22]. Undoubtedly, the high sub-stomatal CO_2_ concentration and low leaf temperature in CIM 18 positively influenced its photosynthetic yield under stress. Conversely, QS21 exhibited stress susceptibility by recording high leaf temperatures and low CO_2_ flow and photosynthetic rate under stress.

Consistent with other studies [23], shoot and root dry weight accumulation was inhibited by stress conditions, with the shoots being more sensitive than the roots. As such, dry shoot weight was used to calculate the different stress indices. High values of STI, DRI and K_2_STI indicated tolerant genotypes. Among the inbred lines evaluated, CIM18 showed moderate tolerance and ranked third, fifth and second in STI, DRI and K_2_STI, respectively. However, QS21 showed great susceptibility to CDHS and was ranked in the bottom 10 for all the indices calculated. Additionally, CIM 18 showed tolerance to heat stress alone in previous laboratory and greenhouse studies [24], and thus could possess genes for CDHS tolerance.

It was hypothesised that comparative analysis of tolerant and susceptible genotypes would result in identification of proteins found under normal growth conditions, those that respond to stress, and those involved in tolerance response. Exposure of CIM18 and QS21 to CDHS in the current study resulted in identification of some proteins with well-known functional roles in plants exposed to abiotic stresses. This allowed us to infer their potential contribution towards the tolerance of CIM118 to CDHS. Other studies have also focused on comparative assessment of transcriptomes differentially expressed by genotypes with contrasting responses to abiotic and biotic stresses. For example, Subramani et al. [25] conducted a comparative analysis of untargeted metabolomics in tolerant and sensitive genotypes of *P. vulgaris* seeds exposed to terminal drought stress. They conducted different pairwise comparisons of drought-tolerant and -sensitive genotypes that were only subjected to drought stress, without exposure to optimum conditions. In their case, the comparisons involved three tolerant and three susceptible genotypes. The comparisons led to successful identification of metabolites associated with drought tolerance. Another study revealed that sweet potato genotypes with contrasting responses to salt stress had distinct differences at the transcription level and translation level even without salt stress [26]. It was reported that proteins that were differentially more abundant in the tolerant type, some of which were absent from the susceptible genotype, were contributing to salt tolerance. Tomato genotypes with contrasting responses to late blight were also used for comparative analysis of their constitutive proteome, without exposing them to stress [27]. They found two defence proteins that were more abundant in the resistant genotype, and these were assumed to be responsible for resistance against late blight.

The proteins identified were involved in numerous biological pathways, which directly and indirectly impact plant protection. The number of proteins micro-sequenced was limited only to those uniquely expressed and upregulated in the tolerant line due to the cost of protein spot identification. Some of the omitted proteins could have likely contributed to tolerance observed in CIM18. Approximately 8.8% more spots were upregulated in CIM18 in comparison to QS21. Subcellular localization revealed that the upregulated proteins were synthesized in the chloroplast and cytoplasm. Therefore, most of these proteins were essential for photosynthesis and energy-related activities, suggesting adaptation to the combined stresses. Similar observations were made in date palm [28] and soybean [29] exposed to CDHS. The identification of proteins and their subcellular locations helps in understanding their physiological function.

A decline in protein abundance reflected cellular damage caused by the two stressors. This included Ribulose- 1,5- bisphosphate carboxylase/oxygenase (Rubisco), Rubisco activase enzymes and other key enzymes of the Calvin cycle, such as phosphoglycerate kinase and fructose–bisphosphate aldolase 1, which catalyses carboxylation. The downregulation of these enzymes is likely responsible for the reduced growth and development observed in QS21 compared to CIM18.

### 3.1. Photosynthesis- and Metabolism-Related Proteins

Rubisco (spot 8611) was one of the proteins associated with photosynthesis that was identified in this study. The increased levels of rubisco in carbon fixation could have been a result of increased energy needs under CDHS. Ribulose biphosphate carboxylase/oxygenase activase is a molecular chaperone responsible for switching Rubisco from an inactive form to an active form. It has been proposed that the reason for the upregulation of these photosynthesis-related proteins is to alleviate damage to Rubisco which is caused by the abiotic stressors [30].

Subunit components of the adenosine triphosphate (ATP) synthase chloroplast protein complex that are involved in ATP synthesis, such as the alpha, gamma and beta subunits, were upregulated in this study. ATP synthase is responsible for the production of ATP from ADP. Adenosine triphosphate is used for various energy-demanding activities during stress, such as protein degradation and biosynthesis, as evidenced by the upregulation of elongation factor 1 alpha, 60S acidic ribosomal protein and HSP70. ATP synthase likely contributed to the extended survival time of plant cells that had become ATP-depleted due to stress [7]. This showed that there was likely activation of ATP-generating pathways under combined stress to meet the increasing demand of ATP to maintain homeostasis. Higher levels of ATP synthase were reported by Loka et al. [31] in cotton subjected to CDHS. The abundance of these proteins and the activities of the Calvin cycle enzymes in CIM18 could have prevented a major reduction in the level of photosynthesis [29].

Photosynthesis depends on Rubisco and the regeneration of RuBP. Results indicated that the Rubisco large chain was upregulated in CIM18 compared to QS21, and this resulted in maintenance of photosynthesis and growth. Although Rubisco occurs abundantly in plant leaf tissue, the combined stress imposed caused its quantity and activity to decrease drastically in susceptible QS21 causing its detection to be difficult. Other reports have shown similar findings under drought or CDHS on non-tolerant plants [30]. The findings support the physiological analysis of CIM18 in another study, where it was ranked among the top 10 inbred lines exhibiting high root to shoot ratio under CDHS [24].

Proteins related to carbohydrate metabolism showed altered expression patterns induced by CDHS. A review by Xu et al. [18] denoted that the concentration of most soluble sugars increased under CDHS and could be used to explain variable tolerance among cultivars. The protein changes related to carbohydrate metabolism were necessary for the adaptation of seedlings to the combination of the two stresses. Carbohydrate metabolism is the main bio-molecular metabolism, and the substrates involved provide energy required by the plant [18,32]. Enzymes related to energy metabolism responded to CDHS in CIM18. Malate dehydrogenase, a protein enzyme involved in carbohydrate synthesis, was upregulated. The abundance of other metabolism-related proteins such as uridine diphosphate glucosyltransferase and glutamine synthetase increased, suggesting a metabolic change in leaves of CIM18 plants under combined stress. Glutathione synthetase showed increased abundance following combined stress in CIM18. These enzymes are important for proline biosynthesis and regulation of bioactivities; their accumulation indicates tolerance to abiotic stressors [8].

It is known that pyruvate phosphate dikinase is a key enzyme in gluconeogenesis and photosynthesis. It reverses the reaction performed by pyruvate kinase in glycolysis and its expression has been associated with tolerance to several abiotic and biotic stressors [33]. Gluconeogenesis requires a lot of energy, which is supplied by the ATP-generating pathways. During exposure to the combined stresses, we observed that there was an increase in the expression of the elongation factor 1-alpha protein, also known as elongation factor thermo-unstable (EF-Tu) protein. The elongation factor thermo-unstable protein was identified as a potential biomarker of heat stress tolerance in soybeans [30]. This protein is an essential component of protein synthesis, and its overexpression greatly improves plant heat stress responses [34].

### 3.2. Heat-Stress-Responsive Proteins

Two heat shock protein families, HSP70 and small HSP 17.8, were identified as stress-related proteins [35]. Heat shock proteins are induced under various stress conditions, but particularly when temperatures exceed 35 °C (temperatures often experienced in the field). In this study, HSPs mediated tolerance to CDHS. These proteins played a significant role in conferring tolerance in CIM18. Several studies have alluded that enhancement of thermo-tolerance is achieved through synthesis of heat shock proteins [14,15]. The findings suggested that drought and heat tolerance was correlated with the accumulation of small heat shock proteins (sHSPs). Small HSP 17.8, uniquely identified in CIM18 in this study, has also been reported by Klein et al. [36] in maize and cardamom [37] subjected to heat stress. The role of HSPs is to stabilize the heat-stressed membranes and proteins while encouraging protein refolding under stress [15]. The tolerant CIM18 inbred line showed increased abundance of HSP70. The function of HSP70 includes protein refolding to maintain cellular homeostasis [38]. Higher expressions of HSP70 have been established in soybean and tomato plants subjected to CDHS [39,40]. Heat shock protein 70 has been widely used as a biomarker under different stressors [41]. However, earlier studies associated HSP with response to heat stress alone instead of other abiotic and biotic stresses.

### 3.3. Antioxidant Proteins

Combined drought and heat stress-induced oxidative stress results in the accumulation of reactive oxygen species (ROS) [38,42]. To scavenge and eliminate these, plants express antioxidant enzymes such as glutathione S transferase (GST). In the current maize study, GST (spot 5404) was upregulated in the tolerant CIM18 line compared with QS21. The accumulation of these antioxidant enzymes constituted part of the detoxification mechanism for excess ROS during oxidative stress. Glutathione S transferase is involved in the glutathione–ascorbate cycle, which is a scavenging system activated in maize to alleviate oxidative stress and enhance drought tolerance. The expression of GST is enhanced by abiotic and biotic stresses [32,43]. The overexpression of protein kinases such as adenylate kinase was likely responsible for the reduction in the accumulation of ROS in the leaves of CIM18. These results corroborate with previous reports, where defence-related proteins such as antioxidant proteins were upregulated under drought stress [43,44]. A study investigating proteomic responses of maize inbred lines with contrasting responses to drought stress also revealed that the tolerant genotype experienced enhanced levels of ROS scavenging enzymes, leading to a higher ROS scavenging ability than in the susceptible genotype [45].

### 3.4. Structural Proteins

The drought stress treatment in this study likely induced actin 1. Actin filaments constitute the cytoskeleton of the plant cell and are responsible for the maintenance of cellular architecture and orientation of organelles [46]. The filaments may also act as osmo-sensors and target potassium ion channels in guard cells for osmoregulation under drought stress. The abundance of this protein in CIM18 could be interpreted as adaptation to drought stress. This corroborates with a report by [47], who reported drought tolerance in barley expressing actin 1.

The potential use of some of the above proteins as biomarkers may be hindered for a few reasons. Before a biomarker can be successfully applied, it must be tested against different genotypes and must be specific, reproducible and sensitive. However, as researchers continue to pursue proteins as biomarkers, they have discovered new techniques to conduct proteomic analysis that are able to overcome challenges posed by the 2-DE gel approach [48].

## 4. Materials and Methods

### 4.1. Plant Materials

Forty-five white quality protein maize inbred lines (Table A2) obtained from the Quality Seed company (Pietermaritzburg, South Africa) and CIMMYT (Harare, Zimbabwe) were assessed for tolerance to CDHS in controlled growth chambers in the Biochemistry and Microbiology Laboratory at the University of Fort Hare, Alice, South Africa. Two inbred lines (QS21 and CIM18) showing contrasting tolerance to CDHS were then used in the proteomic study.

### 4.2. Experimental Design

Forty-five inbred lines were screened for tolerance to CDHS conditions prior to the proteomic experiment. These inbred lines were laid out in a 9 × 5 randomized incomplete block design, with two replications. Each inbred line was replicated three times in each block, and three biological replications (cycles) were performed for each stress condition. The control (no stress imposed) and CDHS experiment lines were grown in separate growth chambers.

In the second experiment, the two selected inbred lines with constating behaviour under CDHS were laid out in a completely randomised design with three replicates and exposed to CDHS treatment. Three pots represented each inbred line per replication, and each experiment was replicated three times over two cycles. In both experiments, plants were grown from seed in pots filled with hygromix growing media (commercial potting mix). Seedlings were thinned to one plant/pot three days after emergence.

### 4.3. Drought and Heat Treatment

The growth chambers were programmed to provide a 14 h/10 h day/night diurnal cycle at an air CO_2_ concentration of 400 µmol mol−1 and 40% relative humidity (RH) [49]. Plants were exposed to drought stress at seven days after emergence by maintaining a field capacity (FC) of approximately 25% for seven days. An SM300 soil moisture meter (Delta T Devices, Cambridge, UK) was used to measure soil moisture content, and plants were irrigated with hygrofert when FC dropped below 25%. Heat stress was imposed on seedlings at the three-leaf stage. Temperature was increased gradually from 25 °C/20 °C (day/night) in 5 °C increments per day to a max of 40 °C/35 °C. The plants were maintained at maximum temperatures for one day. Plants in the high-temperature-regime growth chambers were returned to the control growth chamber (25 °C) for three days to recover [50].

In the control growth chamber, plants were maintained at 75% FC and irrigated once daily with a complete nutrient solution (hygrofert). The growth chambers were programmed to provide a 14 h/10 h day/night diurnal cycle. Air temperature was 25 °C/22 °C, air CO_2_ concentration was maintained at 400 µmol mol−^1^ and RH was 60% for the control and 40% for CDHS. Both experiments were terminated 21 days after initiation [8,18,31].

### 4.4. Data Collection

#### 4.4.1. Physiological Traits

Single-leaf photosynthetic and gaseous exchange measurements were performed with an Integrated Fluorometer and Photosynthesis system (ADC BioScientific Ltd, Hertfordshire, UK). Sub-stomatal CO_2_ (mol mol−1), photosynthetic active radiation (Watts m−2) and photochemical efficiency of photosystem II (ΦPSII = (Fm′ − Fs)/Fm′) measurements were taken in the middle of the third fully developed leaf before, during and after the stress period. The effective quantum yield of PSII was determined on light-adapted leaves during steady-state photosynthesis.

#### 4.4.2. Plant Growth

Shoots and roots were carefully separated, and the latter were thoroughly washed to remove dirt and then oven-dried at 60 °C until a constant weight was obtained. Dry shoot data were used to calculate stress tolerance indices.

### 4.5. Protein Extraction and Quantification

Leaf samples from three plants per genotype and treatment were pooled and ground in liquid nitrogen. Proteins were extracted using the ReadyPrep Protein Extraction Kit (Bio-Rad Laboratories Inc, Sandton, South Africa), followed by sonication and centrifugation at 16,000× *g* for 30 min at 20 °C. The supernatant was collected and standardized to 100 µg using the DC Protein Assay Kit (Bio-Rad), with concentrations determined from a bovine serum albumin (BSA) standard curve.

### 4.6. Protein Clean-Up and Two-Dimensional Electrophoresis

Protein samples were cleaned using the ReadyPrep 2-D Cleanup Kit (Bio-Rad Laboratories Inc, Sandton, South Africa), precipitated, washed and resuspended in rehydration buffer. Proteins (100 µg) were loaded onto 7 cm IPG strips (pH 4–7) and rehydrated overnight. Isoelectric focusing was followed by SDS-PAGE on 12.5% gels. Gels were stained with SYPRO Ruby and visualized using a Uvitec gel documentation system.

### 4.7. Gel Processing and Peptide Analysis

Excised protein spots were destained, dehydrated and subjected to reduction and alkylation using DTT and iodoacetamide. Trypsin digestion was performed overnight at 37 °C. Peptides were extracted using acetonitrile/formic acid, vacuum-dried and cleaned using stage tips for MALDI-TOF analysis.

## 5. Data Analysis

The data presented represents averaged results of three cycles of the 45 inbred lines for every measured parameter. Bartlett’s test was performed to test the homogeneity of error variances for each of the three cycles. The test was significant, and data were combined. Data were found to be normally distributed using Shapiro–Wilk’s test and then subjected to a two-way ANOVA when the conditions and the assumptions of repeated measures were met. The conditions imply sphericity, which means that the variances of the repeated measures are equal and the correlations among the repeated measures are equal. The Tukey HSD test was used to perform post hoc ANOVA comparisons using JMP version 16. Dry shoot weights were used to calculate three tolerance indices using the following formulae.

(1) $$\mathrm{Stress}\;\mathrm{Tolerance}\;\mathrm{Index}\;(\mathrm{STI})=\frac{Y_{p}\times Y_{s}}{Y_{p}2}$$

where Y_s_ and Y_p_ represent shoot dry weight under stress and non-stress conditions, [51].

(2) $$Modified\;Stress\;Tolerance\;Index\;(MSTI)=K2STI,=\left({Y_{S}}^{2})/({{\bar{Y}}_{S}}^{2}\right)$$

where Y_s_ and ${\bar{Y}}_{S}$ represent shoot dry weight under stress and mean yield under stress, respectively [51].

(3) $$Drought\;Resistance\;Index\;(DRI)=\frac{Y_{S}}{Y_{P}}/\frac{{\bar{Y}}_{S}}{{\bar{Y}}_{P}}$$

where Y_s_ and Y_p_ represent shoot dry weight under stress and non-stress conditions, respectively. Also, ${\bar{Y}}_{S}$ and ${\bar{Y}}_{P}$ are the mean yield under stress and non-stress conditions, respectively [51].

Protein spot detection and matching, normalization, quantification and analysis were performed using PDQUEST software 8.0.1 (Bio-Rad). Analytical tools and algorithms in PDQUEST compared the 2-D gels to identify any significant changes in protein expression between tolerant (CIM18) and susceptible (QS21) inbred lines in response to drought and heat stress. Analytical tools in PDQuest helped to identify protein spots of interest. Advanced algorithms in PDQuest identified and matched protein patterns. The 2-D gels for CIM18 and QS21 were selected for comparison and grouped according to treatment. Preset methods for matching and normalization were used. Sophisticated algorithms automated detection and spot matching. The SYPRO Ruby filter provided auto-recognition and removal of background speckles. A spot that occurred in gels of both the tolerant and susceptible genotypes at roughly the same intensity and was identified as being neither up or downregulated by the software was selected and used as a standard for the software’s assessment of whether a protein spot was up- or downregulated. This reference standard was used as the optional control since we negated the need for comparison against the susceptible non-stressed plants (control). Differences in protein abundance between individual gels in the two varieties were validated by Student’s t-test analysis at *p* <0.05. Amino acid sequences for the uniquely identified proteins in the tolerant inbred line were compared with sequences in the database by using a Uniprot-*Zea mays* database. The molecular functions of the identified proteins were classified according to their biological functions using Gene Ontology (GO) annotation from the Blast 2GO software, version 4.

## 6. Conclusions and Recommendations

The results presented here indicate that CDHS inhibited plant growth and biomass accumulation due to an increase in leaf temperatures and reduced sub-stomatal CO_2_ concentration and photosynthetic yield. Inbred lines showing contrasting responses to CDHS were further evaluated by conducting proteomic studies. The tolerant inbred line (CIM18) responded to CDHS by modulating the expression of proteins that have previously been associated with stress response. The upregulation of antioxidant enzymes enhanced the stress defence response of CIM18, in addition to accelerated biosynthesis of proteins such as HSPs and carbohydrate-metabolism-related proteins, all of which might have conferred tolerance to CDHS. The preliminary findings could contribute to understanding the molecular mechanisms of CDHS tolerance in maize. The differentially expressed proteins such as EF-1 alpha and HSP70 can be validated using the ELISA and Western blotting method together with quantitative real-time polymerase chain reaction. It will also be important to find out if the same stress-responsive proteins will be produced at other stages of growth and under field conditions. It is proposed that this proteomics-based knowledge be further investigated before being directly used in the improvement of CDHS tolerance in maize through technologies such as genome editing.

## Figures and Tables

**Table 1:** Mean physiological changes and biomass of the top ten and bottom ten genotypes under combined drought and heat stress.

Rank	PSII		Ci (mol mol^−1^)		T Leaf (°C)		SDW (g)		RDW (g)	
1	QS17	0.69 ^a^	CIM20	9012.00 ^a^	CIM15	35.10 ^a^	QS22	0.17 ^a^	CIM12	0.09 ^a^
2	QS1	0.69 ^ab^	QS23	9012.00 ^a^	QS25	34.60 ^a^	CIM12	0.14 ^ab^	QS22	0.08 ^ab^
3	QS30	0.68 ^a–c^	CIM9	5506.00 ^b^	QS1	34.35 ^ab^	CIM18	0.14 ^a–c^	CIM18	0.08 ^a–c^
4	QS16	0.67 ^a–c^	CIM13 ^b^	5506.00	QS32	34.30 ^ab^	CIM20	0.14 ^a–c^	CIM10	0.08 ^a–c^
5	CIM18	0.67 ^a–c^	CIM21	3935.00 ^c^	QS18	33.95 ^ab^	QS8	0.14 ^a–c^	QS8	0.07 ^a–d^
6	CIM14	0.67 ^a–c^	CIM5	3935.00 ^c^	CIM10	33.80 ^ab^	QS26	0.14 ^a–d^	QS26	0.07 ^a–d^
7	CIM11	0.67 ^a–c^	CIM6	3935.00 ^c^	QS29	33.80 ^ab^	QS18	0.13 ^a–e^	QS18	0.07 ^b–e^
8	CIM12	0.67 ^a–c^	QS3	3935.00 ^c^	CIM16	33.55 ^a–c^	CIM10	0.13 ^b–f^	CIM7	0.06 ^b–f^
9	CIM1	0.66 ^a–c^	QS5	3789.00 ^d^	CIM6	33.50 ^a–c^	QS6	0.12 ^b–f^	QS5	0.06 ^c–g^
10	QS7	0.66 ^a–c^	QS20	3591.00 ^e^	CIM21	33.40 ^a–d^	QS7	0.12 ^b–f^	QS6	0.06 ^c–g^
	Bottom									
1	CIM2	0.60 ^a–e^	CIM16	827.00 ^w^	CIM19	31.10 ^b–j^	QS16	0.09 ^e–i^	QS28	0.04 ^f–h^
2	QS19	0.60 ^a–e^	QS15	827.000 ^w^	QS23	31.05 ^b–j^	QS15	0.09 ^e–i^	CIM2	0.04 ^f–h^
3	QS28	0.57 ^a–e^	QS16	772.000 ^x^	QS21	30.30 ^c–j^	CIM15	0.09 ^e–i^	CIM11	0.04 ^f–h^
4	CIM16	0.53 ^a–e^	CIM17	544.000 ^y^	QS8	30.10 ^d–j^	CIM17	0.09 ^f–i^	CIM13	0.04 ^f–h^
5	CIM21	0.52 ^a–e^	QS21	531.000 ^Z^	CIM7	29.80 ^e–j^	CIM21	0.09 ^f–i^	CIM16	0.04 ^f–h^
6	QS4	0.49 ^b–e^	QS27	531.000 ^z^	QS27	29.35 ^f–j^	QS10	0.09 ^f–i^	QS1	0.04 ^f–h^
7	CIM13	0.49 ^c–e^	CIM1	438.00 ^za^	CIM20	29.00 ^g–j^	QS23	0.09 ^f–i^	QS 10	0.04 ^f–h^
8	CIM19	0.43 ^de^	CIM2	361.00 ^zb^	QS17	28.70 ^h-j^	CIM19	0.08 ^g–i^	QS15	0.04 ^gh^
9	CIM5	0.40 ^e^	QS4	361.00 ^zb^	CIM18	28.60 ^ij^	CIM1	0.08 ^hi^	CIM19	0.04 ^gh^
10	QS5	0.08 ^f^	QS6	−793.00 ^zc^	CIM12	28.30 ^ij^	QS21	0.07 ^i^	CIM1	0.03 ^h^

PSII —ΦPSII = (Fm′ − Fs)/Fm′; Ci—sub-stomatal CO_2_; Tleaf—leaf temperature; SDW—shoot dry weight; RDW—root dry weight. Means followed by the same letter in a column were not significantly different at (*p* ≤ 0.05) according to Tukey’s HSD test.

**Table 2:** Assessment of combined heat and drought stress based on tolerance indices.

INBRED LINE	STI	DRI	K2STI	Inbred Line	STI	DRI	K2STI
CIM1	0.16	0.15	0.09	QS14	0.26	0.17	0.21
CIM18	**0.32**	0.42	**0.58**	QS15	0.23	0.11	0.13
CIM11	0.25	0.44	0.40	QS16	0.18	0.18	0.13
CIM12	0.22	**0.61**	0.40	QS17	0.26	0.28	0.31
CIM13	0.16	0.27	0.14	QS18	0.30	0.30	0.41
CIM14	0.20	0.22	0.17	QS19	0.28	0.26	0.33
CIM15	0.16	0.15	0.09	QS20	0.25	0.18	0.21
CIM16	0.19	0.17	0.13	QS21	0.21	0.28	0.21
CIM17	0.21	0.12	0.12	QS22	**0.43**	**0.54**	**1.07**
CIM10	0.16	0.15	0.09	QS23	0.17	0.14	0.10
CIM2	0.12	0.14	0.05	QS25	0.25	0.23	0.25
CIM20	0.18	0.25	0.15	QS26	0.29	0.32	0.39
CIM21	0.21	**0.54**	0.33	QS27	0.22	0.21	0.18
CIM19	0.14	0.17	0.08	QS28	0.20	0.16	0.14
CIM3	0.21	0.28	0.21	QS29	0.22	0.20	0.19
CIM4	0.22	0.21	0.18	QS30	0.31	0.19	0.31
CIM5	0.28	0.26	0.33	QS32	0.21	0.16	0.15
CIM6	0.26	0.22	0.26	QS4	0.22	0.15	0.16
CIM7	0.30	0.24	0.35	QS5	0.32	0.23	0.38
CIM8	0.27	0.21	0.27	QS6	**0.35**	0.26	**0.47**
CIM9	0.24	0.19	0.20	QS7	0.25	0.29	0.29
QS10	0.20	0.16	0.14	QS8	0.26	0.43	0.41
QS1	0.26	0.22	0.26				

The highest data for each parameter is exhibited in bold. STI—Stress tolerance index; DRI—drought resistance index; K_2_ STI—modified stress tolerance index.

**Table 3:** Identification of differentially responsive proteins in maize leaves subjected to combined drought and heat stress.

Spot #	Protein Names	Accession	% Coverage	Subcellular Location	Function	GO Annotation
4606	Actin-1	sp|P02582|ACT1_MAIZE	9.33	Nucleus (GO:0005634) Cytoskeleton (GO:0005856)	Cytoskeleton organization	GO:0007010
8406	Adenylate kinase, chloroplastic	sp|P43188|KADC_MAIZE	58.11	Plastid; chloroplast (GO:0009507)	Metabolism signalling	GO:0006139 GO:0046940
7403	Glyceraldehyde-3-phosphate dehydrogenase A, chloroplastic	sp|P09315|G3PA_MAIZE	8.933	Plastid; chloroplast (GO:0009507)	Metabolism, photosynthesis	GO:0006006, GO:0006006 GO:0009416, GO:0019253
4607	DIMBOA UDP-glucosyltransferase BX9	sp|B4G072|BX9_MAIZE	36.80	Cytoplasm (GO:0005737)	Metabolism	GO:0008194, GO:0016740 GO:0016757,GO:0047254 GO:0080043, GO:0080044
8509	Glyceraldehyde-3-phosphate dehydrogenase 2, cytosolic	sp|Q09054|G3PC2_MAIZE	48.66	Cytoplasm (GO:0005737)	Metabolism	GO:0006006, GO:0006096
4605	Glutamine synthetase root isozyme 3	sp|P38561|GLNA3_MAIZE	22.47	Cytoplasm (GO:0005737)	Metabolism	GO:0006542
5605	Malate dehydrogenase [NADP], chloroplastic	sp|P15719|MDHP_MAIZE	18.98	Plastid; chloroplast (GO:0009507)	Metabolism	GO:0006099, GO:0006107 GO:0006108, GO:0019674
0405	Glutamine synthetase, chloroplastic	sp|P25462|GLNAC_MAIZE	35.70	Plastid; chloroplast (GO:0009507)	Metabolism	GO:0006542
1606	ATP synthase subunit beta, mitochondrial	sp|P19023|ATPBM_MAIZE	1.989	Mitochondrion; mitochondrion inner membrane (GO:0005743)	Metabolism; ATP metabolism	GO:0046034, GO:0006754 GO:0015986, GO:0042776
5404	Triosephosphate isomerase, cytosolic	sp|P12863|TPIS_MAIZE	22.13	Cytoplasm (GO:0005737)	Metabolism; carbohydrate metabolism	GO:0006094, GO:0006096 GO:0016052
8611	Ribulose bisphosphate carboxylase large chain	sp|P00874|RBL_MAIZE	49.16	Plastid; chloroplast (GO:0009507)	Photosynthesis	GO:0009853, GO:0015977 GO:0015979, GO:0019253
5609	Ribulose bisphosphate carboxylase/oxygenase activase, chloroplastic	sp|Q9ZT00|RCA_MAIZE	19.63	Chloroplast stroma (GO:0009570)	Photosynthesis	GO:0046863, GO:0016887 GO:0005524
4807	Pyruvate, phosphate dikinase 1, chloroplastic	sp|P11155|PPDK1_MAIZE	28.83	Plastid; chloroplast (GO:0009507) Cytoplasm (GO:0005737)	Photosynthesis	GO:0015979
8503	ATP synthase subunit gamma, chloroplastic	sp|P0C1M0|ATPG_MAIZE	42.34	Plastid; chloroplast thylakoid membrane (GO:0009535)	Photosynthesis Proton transmembrane transport	GO:0006754, GO:1902600
3503	60S acidic ribosomal protein P0	sp|O24573|RLA0_MAIZE	19.12	Ribosome (GO:0005840) Cytosolic large ribosomal subunit (GO:0022625)	Protein biosynthesis Stress response to anoxia	GO:0002181 GO:0034059
0406	Elongation factor 1-alpha	sp|Q41803|EF1A_MAIZE	6.711	Cytoplasm (GO:0005737)	Protein biosynthesis	GO:0006412 GO:0006414
5205	17.8 kDa class II heat shock protein	sp|P24632|HSP22_MAIZE	37.20	Cytoplasm (GO:0005737)	Stress response, protein folding Heat response, salt stress response Hydrogen peroxide response Protein oligomerization	GO:0006950, GO:0006457 GO:0009408, GO:0009651 GO:0042542, GO:0051259
4804	Heat shock 70 kDa protein	sp|P11143|HSP70_MAIZE	19.22	Cytoplasm (GO:0005737)	Stress response to heat Protein folding	GO:0009408 GO:0042026
7304	Glutathione S-transferase 3	sp|P04907|GSTF3_MAIZE	16.67	Cytoplasm (GO:0005737)	Stress response to herbicide	GO:0009635

## Data Availability

The data presented in this study are available upon reasonable request from the corresponding author. The data are not publicly available due to [technical/time limitations insert reason here].

## Abbreviations

The following abbreviations are used in this manuscript:

CDHS	Combined drought and heat stress
CIM	CIMMTY
QS	Quality Seed
CO_2_	Carbon dioxide
ATP	Adenosine triphosphate
Rubisco	Ribulose- 1,5- bisphosphate carboxylase/oxygenase
HSP	Heat shock protein
GST	Glutathione S transferase
ROS	Reactive oxygen species
2-D	Two-dimensional

## Appendix A

**Table A1:** A two-way ANOVA of physiological and yield parameters measured under combined drought and heat stress treatments on inbred lines.

Parameters	Inbred	Treatment	Inbred Line × Treatment
Sub-stomatal CO_2_	<0.001	NS	<0.001
Leaf temperature	<0.001	<0.001	<0.001
Photosynthetic yield	<0.001	<0.001	<0.001
Shoot	<0.001	<0.001	<0.001
Root	<0.001	<0.001	<0.001

NS—Not significant.

**Table A2:** Pedigree, drought tolerance and heterotic grouping of quality protein maize inbred lines exposed to combined drought and heat stress.

Line	Pedigree	Drought Tolerance	Heterotic Group
CIM1	(CLQRCWQ50/CML312SR)-2-2-1-BB-1-B-B	-	A
CIM2	[[CML202/CML144]F2-1-1-3-B-1-B*6/[GQL5/[GQL5/[MSRXPOOL9]C1F2-205-1(OSU23i)-5-3-X-X-1-BB]F2-4sx]-11-3-1-1-B*4]-B*5-1-B	drought-tolerant	B
CIM3	[CML141/[CML141/CML395]F2-1sx]-4-2-1-B*4-1-BB-B	drought-tolerant	B
CIM4	[CML144/[CML144/CML395]F2-5sx]-1-3-1-3-B*7-B	drought-tolerant	-
CIM5	[CML144/SNSYNF2[N3/TUX-A-90]-102-1-2-2-BSR-B*4]-B-4-3-B*4-1-B-B	drought-tolerant	B
CIM6	[CML150/CML373]-B-2-2-B*4-4-B-B	drought-tolerant	A
CIM7	[CML159/[CML159/[MSRXPOOL9]C1F2-205-1(OSU23i)-5-3-X-X-1-BB]F2-3sx]-8-1-1-BBB-4-B-B	drought-tolerant	A
CIM8	[CML182/TZMI703]-B-9-1-BB-#-BB-2-B-B	-	B
CIM9	[CML202/CML144]F2-1-1-3-B-1-B*6-2-B	drought-tolerant	B
CIM10	[CML205/CML176]-B-2-1-1-2-B*5-1-B-B	drought-tolerant	B
CIM11	[CML389/CML176]-B-29-2-2-B*4-B	drought-tolerant	B
CIM12	[GQL5/[GQL5/[MSRXPOOL9]C1F2-205-1(OSU23i)-5-3-X-X-1-BB]F2-4sx]-11-3-1-1-B*5-3-B-B	-	B
CIM13	[GQL5/[GQL5/CML202]F2-3sx]-11-4-1-3-B*4-B	-	B
CIM14	[TZMI703/CML176]-B-3-2-B*5-4-B-B	-	B
CIM15	CLQRCWQ50-BB-1-2-B-B	-	B
CIM16	CML176-#-B-2-B	drought-sensitive	B
CIM17	CML181-B-1-5-B-B7	drought-sensitive	-
CIM18	CML182-BB-B	-	-
CIM19	CML264Q-B-1-2-B-B	drought-sensitive	A
CIM20	CML491-B-3-11-B-B	-	A
CIM21	CML492-BB-2-1-B-B	drought-sensitive	B
QS1	K054W	-	F
QS4	V0548W	-	F
QS5	V0298W	-	F
QS6	B0388W	-	F
QS7	EM362W	-	M
QS8	EM583W	-	F
QS10	E625W	-	F
QS14	HM18W	-	O
QS15	HM233W	-	T
QS16	HM238W	-	M
QS17	HM267W	-	F
QS18	HM267W	-	F
QS19	HM268W	-	F
QS20	HM284W	-	H
QS21	HM1472W	-	B
QS22	JM226W	-	H
QS23	JM2341W	-	H
QS25	JM2561W	-	H
QS26	JM2602W	-	H
QS27	JM2621W	-	H
QS28	JM2641W	-	H
QS29	E5	-	G
QS30	E6	-	G
QS32	E27	-	G

CIM—CIMMTY; QS—Quality Seed; -—unknown heterotic group; F—F2834T; M—M37W; O—Obatanpa; T—unknown Tropical; H—Hickory King; B—Brazil; G—Ghana; P—Potch Pearl; A—heterosis similar to Tuxepeno, N3, Reid and Kitale; B—heterosis similar to Ecuador, ETO, SC, Blanco and Lancaster.
